# Inhibition of TGF‐β Increases Bone Volume and Strength in a Mouse Model of Osteogenesis Imperfecta

**DOI:** 10.1002/jbm4.10530

**Published:** 2021-08-03

**Authors:** Benjamin Greene, Ryan J Russo, Shannon Dwyer, Katie Malley, Errin Roberts, Joseph Serrielo, Peter Piepenhagen, Sheila Cummings, Susan Ryan, Christine Zarazinski, Sasidhar Uppuganti, Nikolai Bukanov, Jeffry S Nyman, Megan K Cox, Shiguang Liu, Oxana Ibraghimov‐Beskrovnaya, Yves Sabbagh

**Affiliations:** ^1^ Rare and Neurologic Diseases Research Sanofi Framingham MA USA; ^2^ Global Discovery Pathology Sanofi Framingham MA USA; ^3^ Department of Orthopaedic Surgery Vanderbilt University Medical Center Nashville TN USA; ^4^ Center for Bone Biology Vanderbilt University Medical Center Nashville TN USA; ^5^ Dyne Therapeutics Waltham MA USA; ^6^ Inozyme Pharma Boston MA USA

**Keywords:** BIOCHEMICAL MARKERS OF BONE TURNOVER, OSTEOBLASTS, OSTEOCLASTS, OSTEOGENESIS IMPERFECTA, TGF‐β

## Abstract

Osteogenesis imperfecta (OI), is a genetic disorder of bone fragility caused by mutations in collagen I or proteins involved in collagen processing. Previous studies in mice and human OI bones have shown that excessive activation of TGF‐β signaling plays an important role in dominant and recessive OI disease progression. Inhibition of TGF‐β signaling with a murine pan‐specific TGF‐β neutralizing antibody (1D11) was shown to significantly increase trabecular bone volume and long bone strength in mouse models of OI. To investigate the frequency of dosing and dose options of TGF‐β neutralizing antibody therapy, we assessed the effect of 1D11 on disease progression in a dominant OI mouse model (*col1a2* gene mutation at G610C). In comparison with OI mice treated with a control antibody, we attempted to define mechanistic effects of 1D11 measured via μCT, biomechanical, dynamic histomorphometry, and serum biomarkers of bone turnover. In addition, osteoblast and osteoclast numbers in histological bone sections were assessed to better understand the mechanism of action of the 1D11 antibody in OI. Here we show that 1D11 treatment resulted in both dose and frequency dependency, increases in trabecular bone volume fraction and ultimate force in lumbar bone, and ultimate force, bending strength, yield force, and yield strength in the femur (*p* ≤ 0.05). Suppression of serum biomarkers of osteoblast differentiation, osteocalcin, resorption, CTx‐1, and bone formation were observed after 1D11 treatment of OI mice. Immunohistochemical analysis showed dose and frequency dependent decreases in runt‐related transcription factor, and increase in alkaline phosphatase in lumbar bone sections. In addition, a significant decrease in TRACP and the number of osteoclasts to bone surface area was observed with 1D11 treatment. Our results show that inhibition of the TGF‐β pathway corrects the high‐turnover aspects of bone disease and improves biomechanical properties of OI mice. These results highlight the potential for a novel treatment for osteogenesis imperfecta. © 2021 Sanofi‐Genzyme. *JBMR Plus* published by Wiley Periodicals LLC on behalf of American Society for Bone and Mineral Research.

## Introduction

Osteogenesis Imperfecta (OI) is a rare connective tissue disorder predominantly associated with an increased risk of fractures caused by bone fragility, secondary to altered collagen and decreased bone volume.^(^ ^1^ ^)^ Genetic manifestations include the more common autosomal dominant mutations in genes encoding the triple helical chains of type I collagen, or recessive forms, associated with a variety of genes involved in posttranslational modification of type I collagen.^(^ ^1^, ^2^ ^)^ These loss of function mutations alter the structure of this important connective tissue protein of bone.^(^ ^3^ ^)^ Comprising approximately 40% of the overall volume of bone, type I collagen confers the ability of a hard, brittle mineral phase (nonstoichiometric hydroxyapatite with carbonate) to deform after the onset of loading.^(^ ^4^ ^)^ Defective type I collagen along with decreased bone volume in patients with OI may lead to hundreds of fractures in a lifetime and highlights a clear unmet medical need for therapies that can enhance bone strength, thus leading to a decrease in fracture risk.

Several factors involved in bone remodeling are present locally or in the bone matrix and are released during the remodeling phase. These include growth factors (IGFs, TGF‐βs, BMPs, FGFs, and PDGFs) and cytokines (ILs, TNF, and CSF families). The TGF‐β family of growth factors has its major site of residence in bone.^(^ ^5^ ^)^ Osteoblasts produce TGF‐β, secreting it in a latent form and then depositing it into the bone matrix.^(^ ^6^, ^7^, ^8^ ^)^ During the resorption phase, osteoclasts release and activate TGF‐β from the bone matrix allowing this molecule to influence the bone microenvironment.^(^ ^9^ ^)^ TGF‐β additionally couples bone resorption and formation and recruits osteoprogenitors to the site of bone resorption.^(^ ^10^ ^)^

Impaired TGF‐β signaling has been suggested in patients with OI. A small study in type III patients with OI has documented upregulation of TGF‐β signaling, thus identifying this protein's central role in the pathogenesis of OI.^(^ ^11^ ^)^ Type III patients exhibited increased phospho‐smad2 staining in bone sections, supporting the preclinical finding of increased TGF‐β activity in OI bone.^(^ ^12^ ^)^ Regulation of SMAD phosphorylation was the most significantly upregulated molecular event, whereas TGF‐β activation was identified as the most significant upstream regulator. We previously have shown that administration of an anti‐TGF‐β antibody (1D11) in the jck mouse, a model of renal osteodystrophy characterized by high bone turnover and increased TGF‐β signaling, was able to significantly reduce the elevated pSMAD2/SMAD2 ratio in the femur.^(^ ^13^ ^)^ Genetic and transgenic murine models of TGF‐β signaling have revealed that TGF‐β promotes the pool of osteoprogenitors at the expense of osteoblast differentiation and that there is an inverse relationship between bone matrix quality and signaling through the type II receptor of TGF‐β and downstream effectors (SMAD3).^(^ ^14^ ^)^ These observations led to in vivo, preclinical studies in which inhibition of TGF‐β signaling either by a type‐1 receptor kinase inhibitor or an anti‐TGF‐β antibody in WT mice showed increased bone mass.^(^ ^15^, ^16^ ^)^ It has also been observed that active TGF‐β binds to proteoglycans^(^ ^17^ ^)^ associated with collagen fibrils,^(^ ^18^, ^19^ ^)^ suggesting that collagen plays a role in TGF‐β signaling.

A variety of mouse models have been generated to translate preclinical data to patients with OI. Most prominent among these include the oim/oim (OI type III), Aga2/+ (OI types II and III), BrtlIV/BrtlIV (OI type IV), +/G610C Neo (G610C) (OI types I and III)), Crtap−/− (CRTAP) (OI type VII), and *Col1a1* ^*Jrt/+*^ (OI types III and IV). In this article, we will focus primarily on the *Col1a1* ^*Jrt/+*^, Crtap−/−, and the +/G610CNeo models because of our in‐house work on the +/G610CNeo, as well as discussion of other salient work with anti‐TGF‐β in the Crtap−/− and *Col1a1* ^*Jrt/+*^ models. From a general severity standpoint (measured through bone volume and biomechanical testing), *Col1a1* ^*Jrt/+*^ is the most severe, followed by Crtap−/−, and then +/G610CNeo‐mouse model. Of these three, the CRTAP and G610C models have mutations known to cause OI in humans. However, even though the Col*1a1* ^*Jrt/+*^ does not harbor a human OI mutation, it does suffer from spontaneous fractures, a characteristic it shares with many patients with moderate‐to‐severe OI.^(^ ^20^ ^)^

The TGF‐β signaling pathway was shown to be significantly elevated in the bones of both recessive CRTAP and dominant G610C OI mouse models compared with WT mice, and this elevation contributed to the high bone turnover and low bone mass observed in these OI models.^(^ ^12^ ^)^ Both of these mouse models respond to 1D11therapy, with increases in vertebral and femoral trabecular bone volume fraction (BV/TV) and bending strength of femur mid‐shafts in the CRTAP mouse model.^(^ ^12^ ^)^ In contrast, in the more severe model, Col*1a1* ^*Jrt/+*^, the same effects were not observed. Given the aforementioned beneficial effects of TGF‐β inhibition on the fracture resistance of bones in WT mice and upregulation of TGF‐β signaling in patients with OI, there is potential for a disease‐specific mechanism by which an anti‐TGF‐β blocking antibody can prevent or reduce the number of fractures in patients with OI.

Overall, the preponderance of preclinical results with 1D11 support anti‐TGF‐β therapy in OI, but there are still limitations in our understanding of its pharmacodynamics and mechanism of action. Therefore, we (i) assessed the effect of various dose levels and frequencies of 1D11 treatment on the fracture resistance of OI bone (both femur and vertebra) using the G610C murine model, and (ii) elucidated the mechanism of action of the anti‐TGF‐β antibody, 1D11, in this mouse model via immunofluorescence, immunohistochemistry, histomorphometry, and serum bone biomarker analysis.

## Materials and Methods

### Animals

Animals were provided with food (Auto KF 5 K 52; Lab Diet) and water ad libitum barrier‐ and gang‐housed in pathogen‐free, climate‐controlled facilities with 12‐hour light/dark cycles. The G610C OI (Stock no. 007248; Jackson Labs, hereafter referred to as OI mouse) mouse harbors a mutation in the *Col1a2* gene (*Col1a2tm1.1Mcbr*), which results in a low bone mass^(^ ^21^ ^)^ and a brittle bone phenotype, thus representing a good preclinical model for autosomal dominant OI. Female heterozygous OI mice were used along with their WT littermates (B6.129 background). Male OI mice were not used in these studies because of a contrasting dose‐response curve compared with females. 1D11 appeared more potent in males, increasing trabecular bone volume to near maximal levels at the lowest dose tested (0.3 mg/kg). To simplify data assessment, we limited our analysis to females, but this difference in response will be researched further going forward. Sanofi and Jackson Labs are accredited by the Association for Assessment and Accreditation of Laboratory Animal Care International.

All experiments were approved by Sanofi's Animal Care and Use Committee and included only healthy mice. All mice were randomly assigned to control and treatment groups by birth date across all groups. A dose‐response study with 0.5, 1, 5, and 10 mg/kg of the anti‐TGF‐β antibody (1D11; Sanofi Genzyme), administered ip three times weekly for a total of 8 weeks was evaluated (μCT lumbar n = 10, 9, 9, 10, 9, and 10 for groups 1–6, respectively; μCT femur n = 9, 9, 8, 9, 10, and 9 for groups 1–6, respectively; biomechanics lumbar n = 10, 9, 9, 10, 10, and 10 for groups 1–6, respectively; and biomechanics femur n = 10, 9, 9, 10, 9, and 9 for groups 1–6, respectively). A dose‐frequency study at a dose of 5 mg/kg of 1D11, administered ip either three times weekly, one time weekly, one time every 2 weeks, or one time every 4 weeks, for a total of 12 weeks was evaluated (μCT lumbar n = 8, 5, 5, 8, 8, and 8 for groups 1–6, respectively; μCT femur n = 8, 5, 5, 8, 8, and 8 for groups 1–6, respectively; biomechanics lumbar n = 8, 5, 5, 7, 8, and 8 for groups 1–6, respectively; and biomechanics femur n = 8, 5, 5, 8, 8, and 9 for groups 1–6, respectively). A single dose study with 5 mg/kg of 1D11 administered ip three times weekly for a total of 8 weeks was evaluated (dynamic histomorphometry lumbar n = 7, 4, and 6 for groups 1–3, respectively). Mice were additionally administered an ip injection of the fluorochrome compound Alizarin Red at a dose of 40 mg/kg, ip and calcein at 15 mg/kg 7 and 2 days before necropsy, respectively. A short‐term study with a total of four doses of 5 mg/kg of 1D11, administered ip over 1 week was done for mechanism of action studies (n = 3, 3, and 6 for groups 1–3, respectively). All dosing in mice was initiated at 8 to 11 weeks of age and included separate groups of WT and OI mice receiving the isotype antibody, control (13C4; Sanofi Genzyme) at either the highest dose or highest frequency for the above studies. Studies with 13C4 have shown no effect on any bone parameters in G610C mice compared with saline control (data not shown). All mice were test naïve before treatment, and no adverse events were noted in these studies in any group with any treatment. Following each treatment regimen as described above, mice were euthanized and serum and bone were collected. The spine was harvested and the fourth and sixth lumbar bones were separated, cleaned, and stored in 40% ethanol or wrapped in saline‐soaked gauze and kept at −20°C for histomorphometry and μCT/biomechanical testing, respectively. Both left and right femurs were also harvested and stored in 40% ethanol or wrapped in saline‐soaked gauze and kept at −20°C for histomorphometry and μCT/biomechanical testing, respectively.

### Immunofluorescence

Paraffin slides were dewaxed and rehydrated followed by antigen retrieval using sodium citrate solution (pH 9.9; Dako) and placed in a pressure cooker for 15 minutes. Cooled slides were blocked in 3% BSA/PBS for 20 minutes. The l4 or femur sections were incubated with rabbit polyclonal anti‐runt–related transcription factor (RUNX2) antibody, 1:100 dilution (ab23981; Abcam) or with rabbit monoclonal anti‐alkaline phosphatase (ALP), tissue nonspecific antibody, 1:100 dilution (ab108337; Abcam) in 3% BSA/PBS. Donkey anti‐Rabbit IgG (H + L) highly cross‐adsorbed secondary antibody, Alexa Fluor Plus 594 (A32754; Thermo Fisher Scientific) was used to detect primary antibodies.

### Biomarkers

Blood samples were collected via cardiac puncture immediately before necropsy at the end of the study. Serum osteocalcin (OCN) and CTx were measured using the Mouse Osteocalcin EIA Kit (Catalog no. BT‐470; Alfa Aesar) and the RatLaps EIA Kit (Catalog no. AC‐06F1; Immunodiagnostic Systems Ltd), respectively, according to the manufacturer's protocol.

### μCT evaluations

After securely aligning the long axis of each femur or each l6 vertebra (VB) in a specimen tube (od = 6 mm), the midpoint of the diaphysis (length = 1.86 mm), the distal femur metaphysis including part of the growth plate (length = 3.72 mm), and cranial‐to‐caudal endplates (length = ~2.88 mm) were scanned by μCT (Scanco50; Scanco Medical AG) to provide gray‐scale images with an isotropic voxel size of 6 μm (femur) or 12 μm (VB). The scan settings were: (i) peak voltage = 70 kVp with a tube current = 0.114 mA, integration time = 1000 ms, and sampling rate = 1160 samples per 500 projections per 360° rotation of the sample tube (femur); and (ii) peak voltage = 55 kVp with a tube current = 0.200 mA, integration time = 1200 ms, and sampling rate = 494 samples per 1000 projections per 360° rotation (VB). To minimize the undue influence of beam hardening on the attenuation of X‐rays, an aluminum filter (thickness of 0.1 mm) was placed in the X‐ray path. Also, the manufacturer's beam‐hardening correction was used. Following the scans, the images were reconstructed and evaluated using Scanco evaluation software version 6.6. For selecting the ROI for evaluation, we tightly fit contours to the periosteal and endosteal surface of the femur diaphysis, placed contours approximately 2 voxels into the marrow space from the endosteal surface of the distal femur metaphysis (starting 2.52‐mm proximal from the primary spongiosa within the growth plate and spanning 2.10 mm in length), or drew contours within the centrum of the VB and between the endplates. Because the scanner is routinely calibrated to the manufacturer‐provided standard hydroxyapatite (HA) phantom (200–1200 mg HA/cm^3^), the bone tissue in the reconstructed and filtered images (Gaussian ς = 0.8 and Gaussian support = 1.0 to suppress image noise) was segmented from air and marrow using global thresholds of 936.7 mgHA/cm^3^ (cortical bone of femur diaphysis), 417.3 mgHA/cm^3^ (trabecular bone of femur metaphysis and trabecular bone of VB). Standard Scanco evaluation algorithms were used to determine the structural properties of the diaphysis (eg, cortical thickness, cross‐sectional area, moment of inertia), architectural properties of trabecular bone (eg, bone volume fraction, trabecular thickness, trabecular number), and the tissue mineral density of each compartment. Technicians were blinded during image capturing and analysis as to both genotype and group.

### Biomechanical testing

With the anterior side facing down and the medial side facing forward, the midpoint of femoral diaphysis was loaded‐to‐failure at 3 mm/min using a three‐point bending fixture (lower span of 8 mm). The material testing system (8800 DynaMight; Instron) recorded the force from the load cell (maximum capacity of 100 N) and the deflection of the bone from the linear variable displacement transducer attached to the actuator. Hydration of the femur was maintained throughout testing. Identifying the yield point by a 15% loss in secant stiffness, the structural‐dependent properties (stiffness, yield force, ultimate force, and work‐to‐fracture) and postyield displacement were determined from the resulting force versus displacement curve. Using the minimum moment of inertia (I_min_ corresponding to bending about anterior‐to‐posterior plane), the distance from the centroid to the outermost surface in the anterior–posterior direction (C_min_), and cross‐sectional area of the cortex (Ct.Ar), we estimated several material properties as follows: modulus (stiffness × span^3^/48/I_min_), yield stress and peak stress (yield or ultimate force × span/4 × C_min_/I_min_), and toughness (3 × work‐to‐fracture/Ct.Ar/span).^(^ ^22^ ^)^ Each hydrated VB was loaded‐to‐failure at 3 min/min in compression between two rigid stainless‐steel plates: one attached to the actuator of the material testing system that loaded the cranial end of the VB, and one attached to the load cell (100 N) with a moment‐relief setup to ensure axial transmission of the loading force.^(^ ^23^ ^)^ The VB strength was determined by the ultimate force endured by the bone. Technicians were blinded during image capturing and analysis as to both genotype and group.

### Histomorphometry

Lumbar vertebrae (l4) were fixed in 40% alcohol and then routinely processed and embedded in methyl methacrylate. Six 5‐μm serial sections were taken (RM2255; Leica Biosystems) at the midpoint. One slide was stained with von Kossa and used for structural measurements using Bioquant Osteo Histomorphometric program. Another slide was left unstained and was used for dynamic measurements using the same quantitative system mentioned above. For each of these measurements, l4 vertebrae were analyzed using a 1500 × 1800‐μm ROI approximately 150 μm from the growth plate. In addition, immunohistochemistry for tartrate‐resistant acidic phosphatase (TRAP; Sigma‐Aldrich) was performed on methyl‐methacrylate sections of l4 vertebrae. These were analyzed using a 1200 × 500‐μm ROI, approximately 50 μm from the growth plate. Active osteoclasts were manually counted 20 times in a grid‐like pattern along the ROI. The Bioquant Osteo Histomorphometric program was used to quantitate TRAP‐stained osteoclasts.

### Statistical analysis

Statistical significance was determined by one‐way ANOVA followed by a Dunnett's post hoc test using GraphPad Prism version 8.0.2. Values of *p* ≤ 0.05 were considered significant.

## Results

### μCT and biomechanical testing

#### Lumbar vertebral body, femur metaphysis, and femur diaphysis

##### Dose response

**Fig. 1:**
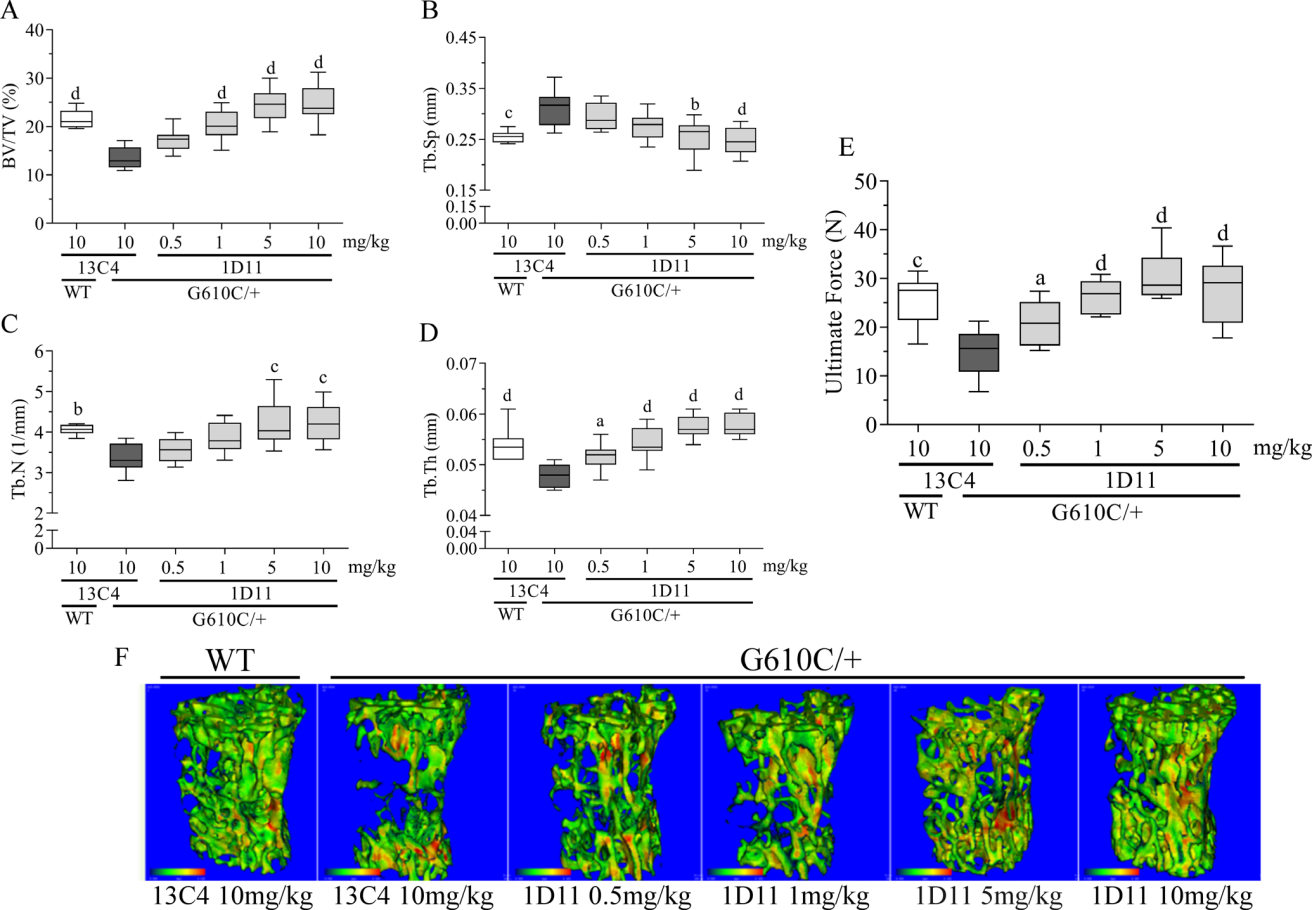
Anti‐TGF‐β antibody 1D11 induces robust, dose‐related increases in lumbar bone (l6) trabecular parameters and strength mice with osteogenesis imperfecta (OI). (*A*) Bone volume, (*B*) trabecular separation, (*C*) trabecular number, (*D*) trabecular thickness, and trabecular total mineral density all via μCT imaging, and (*E*) peak force measured via biomechanical compression test of OI and WT mice. μCT lumbar n = 10, 9, 9, 10, 9, and 10 for group 1–6, respectively. Biomechanics lumbar n = 10, 9, 9, 10, 10, and 10 for groups 1–6, respectively. Mean ± SD and asterisk(s) denote statistically significant difference compared with OI 13C4, 10 mg/kg. ^a^0.01 < *p* < 0.05. ^b^0.001 < *p* < 0.01. ^c^0.0001 < *p* < 0.001. ^d^ *p* < 0.0001. BV/TV = bone volume fraction; Tb.N = trabecular number; Tb.Sp = trabecular spacing; Tb.Th = trabecular thickness.

**Fig. 2:**
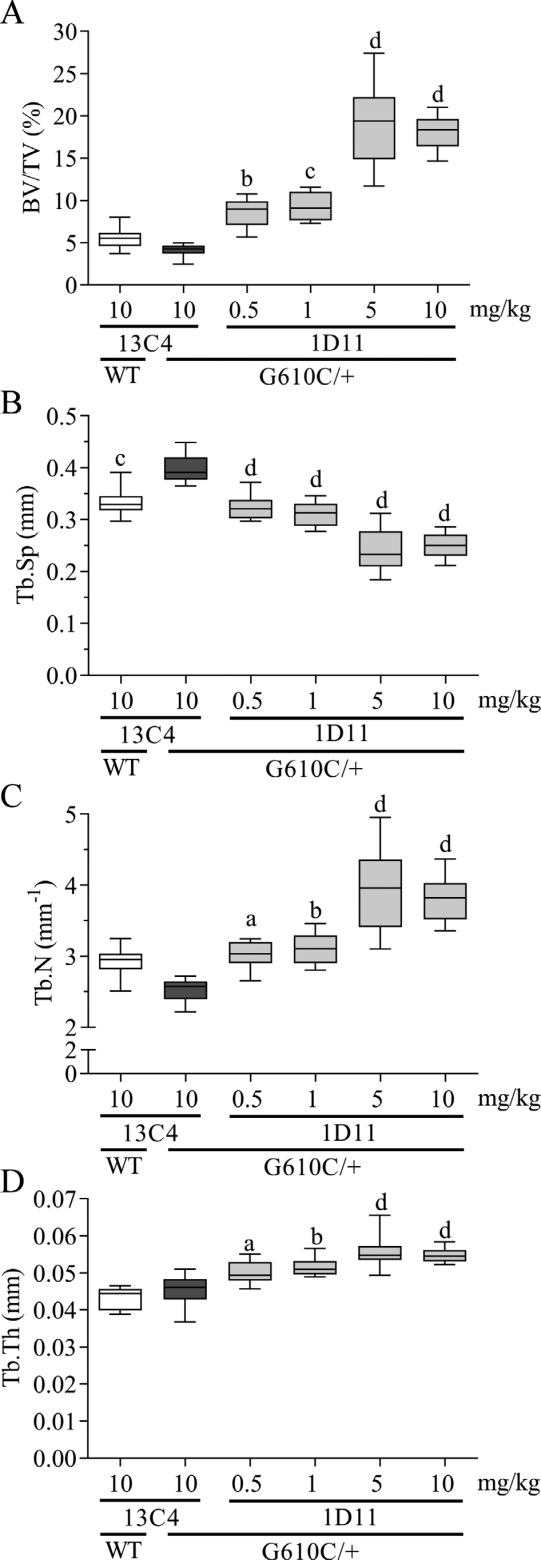
Anti‐TGF‐β antibody 1D11 induces robust, dose‐related increases in femoral bone trabecular parameters in mice with osteogenesis imperfecta (OI) measured by μCT imaging. (*A*) Bone volume, (*B*) trabecular separation, (*C*) trabecular number, and (*D*) trabecular thickness measured in femoral metaphysis of OI and WT mice. μCT femur n = 9, 9, 8, 9, 10, and 9 for groups 1–6, respectively. Mean ± SD and asterisk(s) denote statistically significant difference compared with OI 13C4, 10 mg/kg ip. ^a^0.01 < *p* < 0.05. ^b^0.001 < *p* < 0.01. ^c^0.0001 < *p* < 0.001. ^d^ *p* < 0.0001. BV/TV = bone volume fraction; Tb.N = trabecular number; Tb.Sp = trabecular spacing; Tb.Th = trabecular thickness.

1D11‐treatment robustly, and in a dose‐dependent fashion, increased BV/TV (Fig. 1*A*.) in the LV body (l6) and femoral metaphysis (Fig. 2*A*.) of OI mice. This coincided with a marked, and again, dose‐dependent increase in ultimate force of l6 (Fig. 1*E*). Dose‐dependent increases in trabecular number and thickness with a corresponding decrease in separation were also noted in l6 (Fig. 1*B*–*D*) and femoral metaphysis (Fig. 2*B*–*D*). Overall, the minimum effective dose was 0.5 mg/kg, whereas maximal effect was reached at 5 mg/kg, all at three weekly doses.

**Fig. 3:**
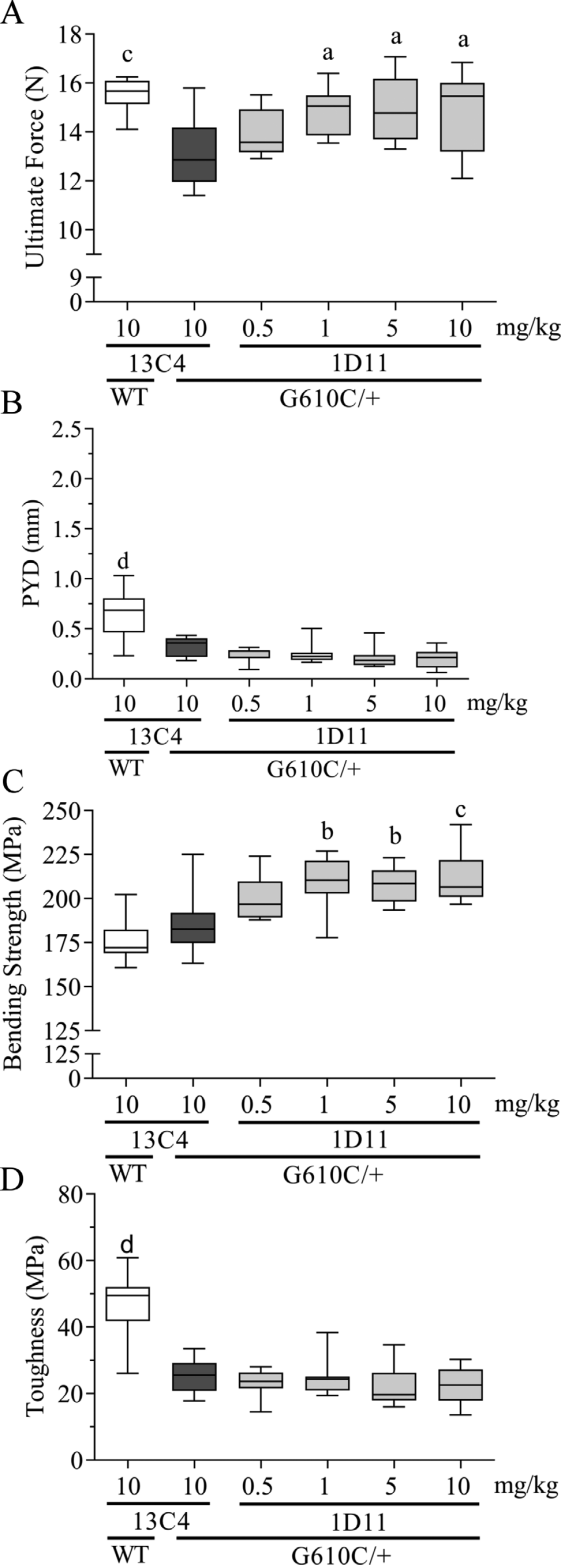
Anti‐TGF‐β antibody 1D11 induces dose‐related increases in femoral strength (*A*,*C*), but has no effect on postyield displacement (PYD) and toughness (*B*,*D*) in mice with osteogenesis imperfecta (OI) measured via a three‐point bending test. Biomechanics femur n = 10, 9, 9, 10, 9, and 9 for groups 1–6, respectively. Mean ± SD and asterisk(s) denote statistically significant difference compared with OI 13C4, 10 mg/kg ip. ^a^0.01 < *p* < 0.05. ^b^0.001 < *p* < 0.01. ^c^0.0001 < *p* < 0.001. ^d^ *p* < 0.0001.

Relative to OI mice treated with the 13C4 control‐isotype antibody, 1D11 treatment induced a dose‐dependent increase in both ultimate force endured by the femoral diaphysis and bending strength (Fig. 3*A*,*C*) of cortical bone. Additionally, in the OI mice, the modulus, yield force, and yield strength (femur diaphysis) all increased in a dose‐dependent fashion (Table 1), whereas toughness and postyield displacement did not increase with 1D11 treatment (Fig. 3*B*,*D*). The only cortical bone parameter that was significantly elevated was cortical thickness at a 5‐mg/kg dose (Table 1). Overall, the minimum effective dose was 0.5 mg/kg, whereas maximal effect was reached at 1 to 5 mg/kg, all at three weekly doses.

**Table 1:** Effect of Treatment on Cortical Bone and Strength in the Femur of WT and OI Mice

Property	WT 13C4	OI 13C4	OI 1D11	OI 1D11	OI 1D11	OI 1D11
	10 mg/kg	10 mg/kg	0.5 mg/kg	1 mg/kg	5 mg/kg	10 mg/kg
Cortical (diaphysis)						
Cortical bone area (mm^2^)	0.744 ± 0.030 ^c^	0.654 ± 0.042	0.671 ± 0.036	0.682 ± 0.020	0.686 ± 0.037	0.670 ± 0.044
Total cross‐sectional area (mm^2^)	1.70 ± 0.068 ^c^	1.40 ± 0.080	1.37 ± 0.073	1.39 ± 0.051	1.40 ± 0.055	1.39 ± 0.089
Minimum moment of inertia (mm^4^)	0.110 ± 0.011 ^c^	0.080 ± 0.008	0.078 ± 0.008	0.081 ± 0.007	0.081 ± 0.008	0.079 ± 0.009
Average cortical thickness (mm)	0.163 ± 0.005 ^b^	0.153 ± 0.007	0.160 ± 0.007	0.161 ± 0.006	0.162 ± 0.004 ^a^	0.159 ± 0.010
Biomechanical						
Stiffness (N/mm)	93.0 ± 18.6	96.8 ± 13.5	101 ± 13.4	110 ± 7.99	112 ± 11.7	106 ± 12.3
Modulus (GPa)	9.03 ± 1.70 ^c^	12.9 ± 1.46	13.8 ± 0.914	14.6 ± 0.832 ^b^	14.8 ± 0.989 ^b^	14.4 ± 0.594 ^a^
Yield force (N)	12.2 ± 1.49	11.7 ± 1.29	13.0 ± 1.49	13.5 ± 1.02 ^a^	13.5 ± 1.02 ^a^	13.5 ± 1.40 ^a^
Yield strength (MPa)	138 ± 20.9 ^b^	165 ± 13.7	187 ± 17.7 ^a^	191 ± 17.5 ^b^	189 ± 12.9 ^a^	195 ± 21.1 ^b^
Work‐to‐fracture (N*mm)	8.86 ± 2.19 ^c^	4.23 ± 0.821	3.89 ± 0.612	4.05 ± 0.787	3.77 ± 0.928	3.71 ± 0.926

Mean ± SD and asterisk(s) denote statistically significant difference compared with OI 13C4, 10 mg/kg.

mg HA = mg hydroxyapatite; OI = osteogenesis imperfecta.

^a^ 0.01 < *p* < 0.05.

^b^ 0.001 < *p* < 0.01.

^c^ *p* < 0.0001.

##### Dose frequency

**Fig. 4:**
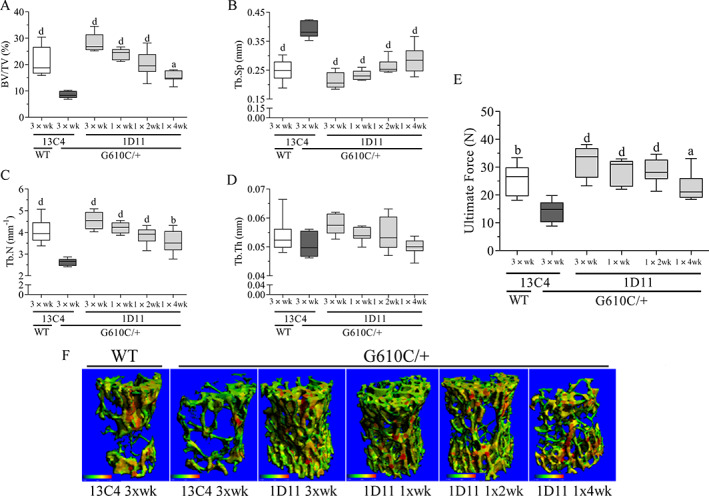
Anti‐TGF‐β antibody 1D11 induces robust and durable dose‐frequency–related increases in lumbar (l6) trabecular parameters and strength in mice with osteogenesis imperfecta (OI). (*A*) Bone volume, (*B*) trabecular separation, (*C*) trabecular number, (*D*) trabecular thickness (no statistical change), (*E*) trabecular total mineral density all measured with μCT imaging, and (*F*) ultimate force measured via biomechanical compression test of OI and WT mice. μCT lumbar n = 8, 5, 5, 8, 8, and 8 for groups 1–6, respectively; biomechanics lumbar n = 8, 5, 5, 7, 8, and 8 for groups 1–6, respectively. Mean ± SD and asterisk(s) denote statistically significant difference compared with OI 13C4, three times weekly. ^a^0.01 < *p* < 0.05. ^b^0.001 < *p* < 0.01. ^d^ *p* < 0.0001. Dosing regimen: 3 × wk = three weekly doses; 1 × wk = one weekly dose; 1 × 2 wk = one dose every other week; 1 × 4 wk = one dose every 4 weeks; all doses 5 mg/kg ip. BV/TV = bone volume fraction; Tb.N = trabecular number; Tb.Sp = trabecular spacing; Tb.Th = trabecular thickness.

**Fig. 5:**
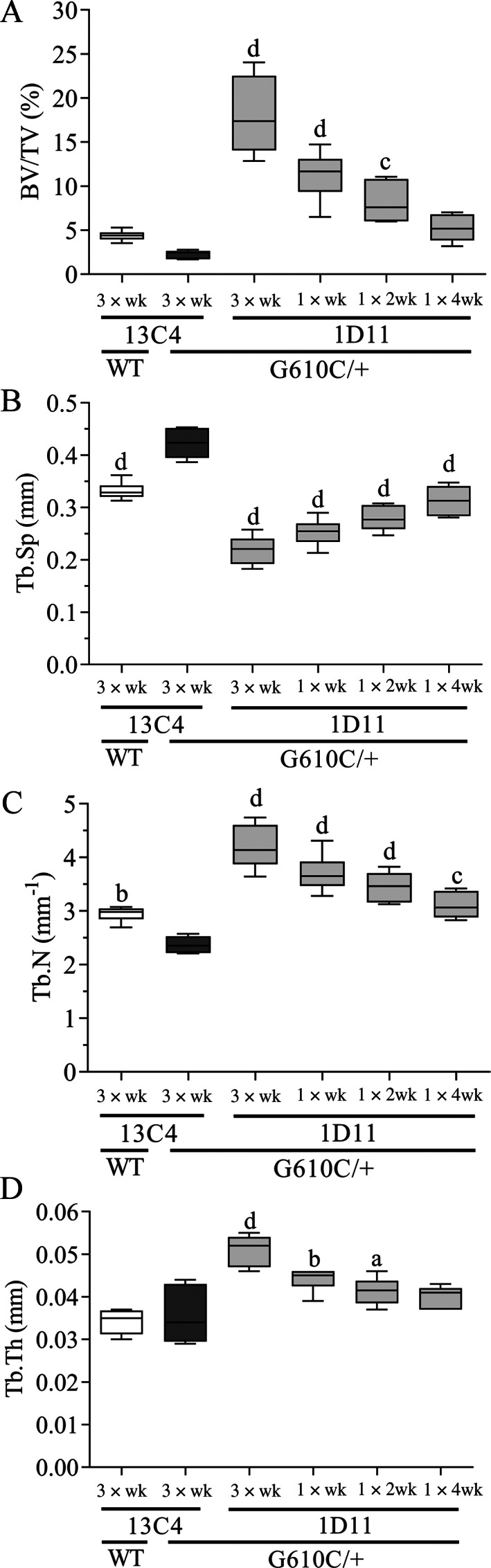
Anti‐TGF‐β antibody 1D11 induces robust and durable dose‐frequency–related increases in femoral bone trabecular parameters in mice with osteogenesis imperfecta (OI) measured by μCT imaging. (*A*) Bone volume, (*B*) trabecular separation, (*C*) trabecular number, and (*D*) trabecular thickness measured in femoral metaphysis of WT and OI mice. μCT femur n = 8, 5, 5, 8, 8, and 8 for groups 1–6, respectively. Mean ± SD and asterisk(s) denote statistically significant difference compared with OI 13C4, 10 mg/kg ip. ^a^0.01 < *p* < 0.05. ^b^0.001 < *p* < 0.01. ^c^0.0001 < *p* < 0.001. ^d^ *p* < 0.0001. Dosing regimen: 3 × wk = three weekly doses; 1 × wk = 1 weekly dose; 1 × 2 wk = one dose every other week; 1 × 4 wk = one dose every 4 weeks; all doses 5 mg/kg ip. BV/TV = bone volume fraction; Tb.N = trabecular number; Tb.Sp = trabecular spacing; Tb.Th = trabecular thickness.

1D11 treatment robustly, and in a dose frequency‐dependent fashion, increased BV/TV (Fig. 4*A*) in l6 and femoral metaphysis (Fig. 5*A*) of OI mice. With this marked increase in BV/TV, ultimate force (Fig. 4*F*) of the l6 vertebral body also increased in a frequency dependent manner. Dose frequency dependent increases in trabecular number, and a corresponding decrease in separation, also occurred in l6 and femoral metaphysis (Figs. 4*B*,*C* and 5*B*,*C*). Additionally, a dose frequency‐dependent increase in trabecular thickness was observed in the femoral metaphysis (Fig. 5*D*). Overall, the minimum effective dose frequency was one dose every 4 weeks, whereas maximal effect was reached at three weekly doses.

**Fig. 6:**
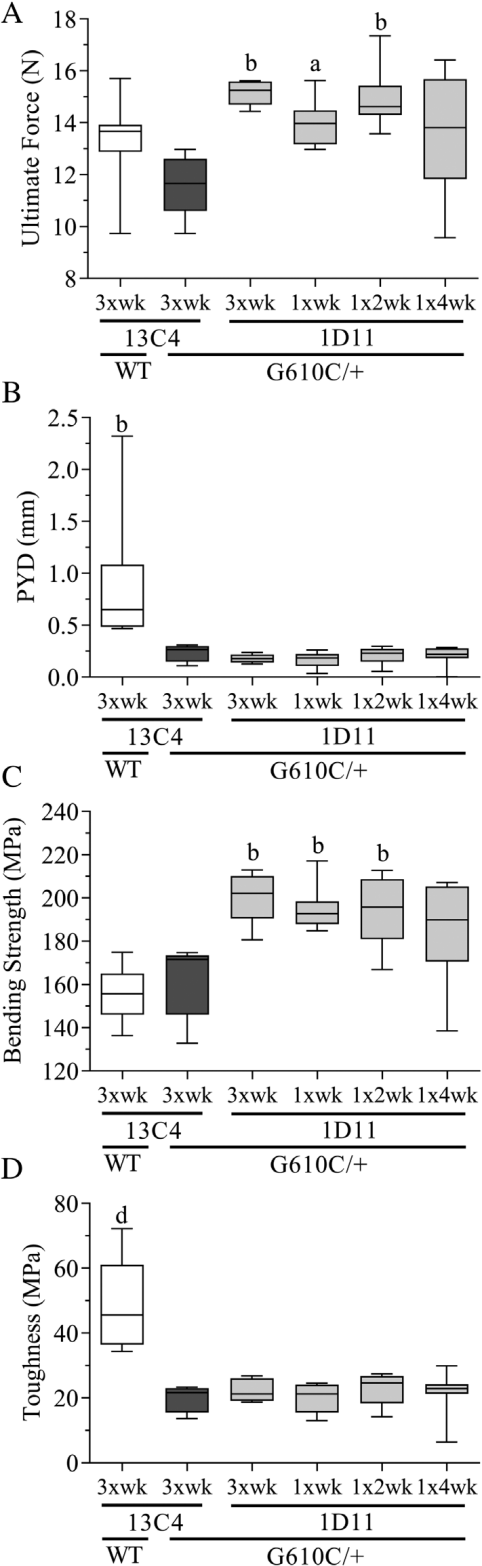
Anti‐TGF‐β antibody 1D11 induces durable increases in femoral strength (*A*,*C*), but has no effect on postyield displacement and toughness (*B*,*D*) in mice with osteogenesis imperfecta (OI) measured via three‐point bending test. Biomechanics femur n = 8, 5, 5, 8, 8, and 9 for groups 1–6, respectively. Mean ± SD and asterisk(s) denote statistically significant difference compared with OI 13C4, 10 mg/kg ip. ^a^0.01 < *p* < 0.05. ^b^0.001 < *p* < 0.01. ^c^0.0001 < p < 0.001. ^d^ *p* < 0.0001. Dosing regimen: 3 × wk = three weekly doses; 1 × wk = 1 weekly dose; 1 × 2 wk = one dose every other week; 1 × 4 wk = one dose every 4 weeks; all doses 5 mg/kg ip. BV/TV = bone volume fraction; Tb.N = trabecular number; Tb.Sp = trabecular spacing; Tb.Th = trabecular thickness. [Correction added on 11 August 2021, after first online publication: Figure 6 has been replaced]

1D11 treatment also induced increases in both ultimate force and bending strength (Fig. 6*A*,*C*) in the femoral diaphysis of OI mice in comparison with OI mice‐treated 13C4 control. Additionally, in the OI mice, yield force and yield strength (femur diaphysis) both increased at several dose frequencies (Table 2), whereas toughness and postyield displacement did not increase with 1D11 treatment (Fig. 6*B*,*D*). A significant increase in cortical bone area was noted (Table 2), but as above, most cortical parameters exhibited no effect, including minimum moment of inertia. Overall, the minimum effective dose frequency was one dose every 4 weeks, whereas maximal effect was reached at a dose frequency of one dose every 2 weeks.

**Table 2:** Effect of Treatment on Cortical Bone and Biomechanics in the Femur of WT and OI Mice

Property	WT 13C4	OI 13C4	OI 1D11	OI 1D11	OI 1D11	OI 1D11
	5 mg/kg	5 mg/kg	5 mg/kg	5 mg/kg	5 mg/kg	5 mg/kg
	3 × wk	3 × wk	3 × wk	1 × wk	1 × 2 wk	1 × 4 wk
Cortical (Diaphysis)						
Cortical bone area (mm^2^)	0.700 ± 0.047	0.645 ± 0.027	0.719 ± 0.018 ^a^	0.702 ± 0.032 ^a^	0.704 ± 0.031 ^a^	0.690 ± 0.050
Total cross‐sectional area (mm^2^)	1.72 ± 0.098 ^c^	1.43 ± 0.040	1.49 ± 0.031	1.45 ± 0.084	1.50 ± 0.063	1.44 ± 0.076
Minimum moment of inertia (mm^4^)	0.106 ± 0.012 ^c^	0.081 ± 0.004	0.089 ± 0.004	0.083 ± 0.008	0.089 ± 0.008	0.085 ± 0.008
Average cortical thickness (mm)	0.151 ± 0.009	0.151 ± 0.010	0.158 ± 0.006	0.154 ± 0.010	0.161 ± 0.005	0.158 ± 0.005
Biomechanical						
Stiffness (N/mm)	90.1 ± 12.5	90.3 ± 13.8	109 ± 18.4	101 ± 9.15	106 ± 7.29	101 ± 16.4
Modulus (GPa)	9.14 ± 1.41 ^a^	11.9 ± 1.80	13.2 ± 2.22	13.0 ± 1.26	12.9 ± 1.39	12.7 ± 1.99
Yield force (N)	10.8 ± 2.28	11.2 ± 1.47	14.7 ± 1.12 ^a^	13.3 ± 1.06	14.7 ± 1.19 ^b^	13.2 ± 2.46
Yield strength (MPa)	126 ± 21.1 ^a^	156 ± 21.5	195 ± 19.9 ^a^	185 ± 10.7 ^a^	191 ± 17.3 ^a^	180 ± 23.7
Work‐to‐fracture (N*mm)	8.88 ± 2.33 ^c^	3.42 ± 0.85	3.83 ± 0.437	3.36 ± 0.873	4.10 ± 0.892	3.67 ± 1.26

Mean ± SD and asterisk(s) denote statistically significant difference compared with OI 13C4 3 × wk, 5 mg/kg, ip.

mg HA = mg hydroxyapatite; OI = osteogenesis imperfecta.

^a^ 0.01 < *p* < 0.05.

^b^ 0.001 < *p* < 0.01.

^c^ *p* < 0.0001.

### Histomorphometric analysis

**Fig. 7:**
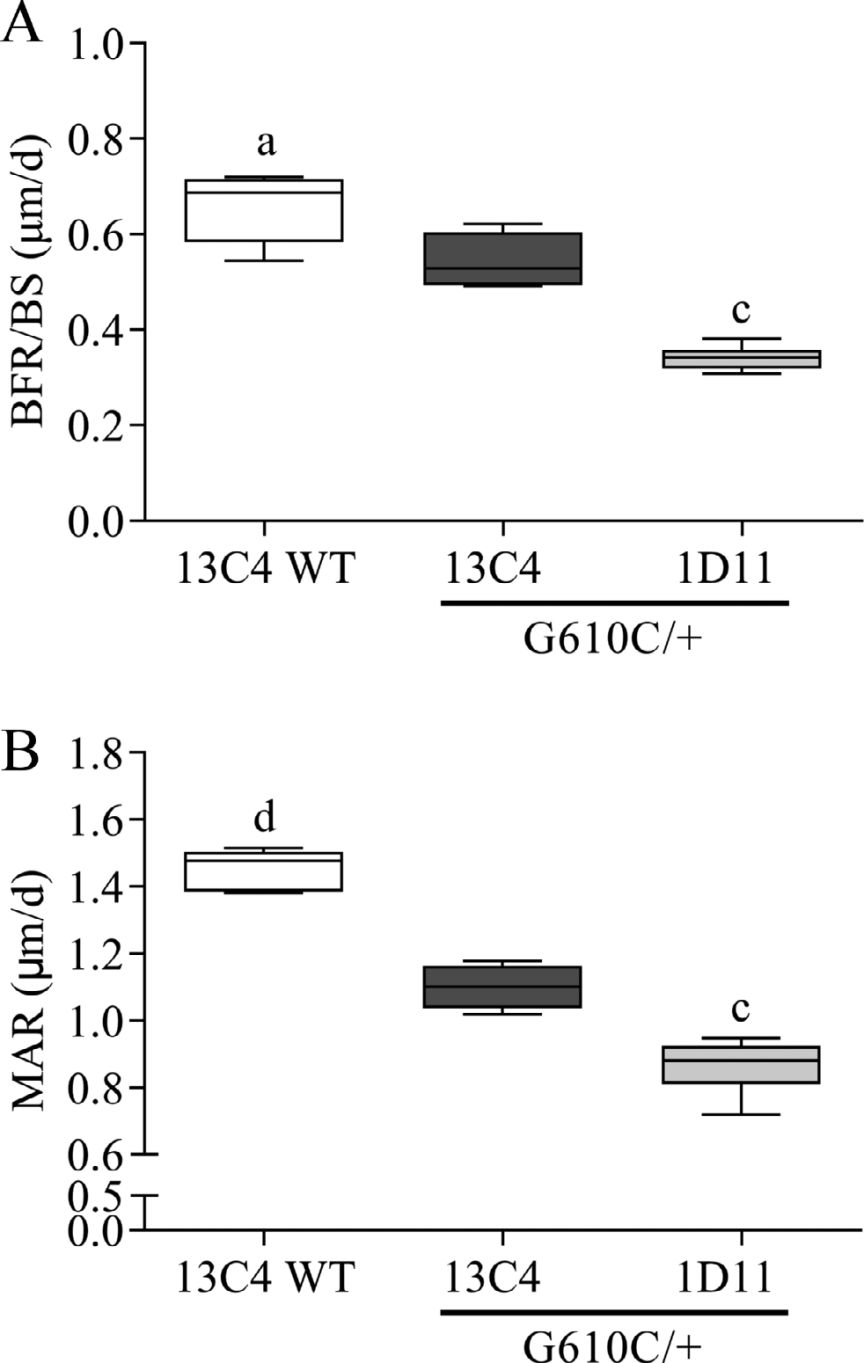
Anti‐TGF‐β antibody 1D11 decreases bone formation rate/body surface (BFR/BS) and mineral apposition rate (MAR) in mice with osteogenesis imperfecta (OI) relative to 13C4 control at three‐times weekly dosing for 8 weeks in OI mice. BFR/BS and MAR are lower in OI compared with WT mice, both treated with 13C4 control, n = 8, 5, 5, and 8 for groups 1–4, respectively. Mean ± SD and asterisk(s) denote statistically significant difference compared with OI 13C4. ^a^0.01 < *p* < 0.05. ^b^0.001 < *p* < 0.01. ^c^0.0001 < *p* < 0.001. ^d^ *p* < 0.0001. All doses 5 mg/kg ip.

1D11 treatment robustly, and in a dose frequency‐dependent fashion, increased BV/TV (Supplementary Information Fig. S1 *A*) in the lumbar vertebral body (l4) of OI mice. Dose frequency‐dependent increases in trabecular thickness and separation also occurred in l4, along with significant increases in trabecular number (Supplementary Information Fig. S1 *B*–*D*). These data confirm the μCT and biomechanical data reported earlier (Fig. 4). Overall, the minimum‐effective dose frequency was one dose every 4 weeks, whereas maximal effect was reached at three weekly doses for most parameters. Relative to OI mice treated with 13C4, 1D11 treatment at 5 mg/kg ip with three weekly doses decreased both the bone formation rate/body surface (BFR/BS) and the mineral apposition rate (MAR; Fig. 7*A*,*B*).

### Mechanism of action of anti‐TGF‐β antibody on osteoblasts and osteoclasts

To better understand the mechanism of action of the anti‐TGF‐β antibody on osteoblast and osteoclast function, the expression of specific markers was evaluated in bone sections of OI mice treated with 1D11 antibody.

**Fig. 8:**
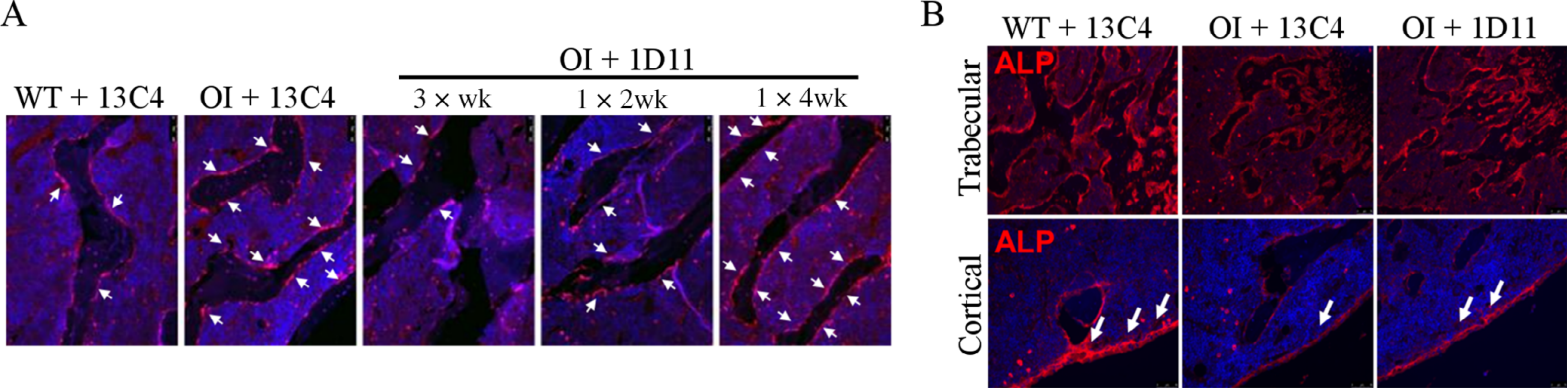
Anti‐TGF‐β antibody 1D11 restores osteoblast differentiation as evidenced by effects on runt‐related transcription factor 2 (RUNX2) and alkaline phosphatase (ALP). Immunohistochemistry performed in lumbar bone (*A*) shows increased RUNX2 expression on the trabecular bone surface in osteogenesis imperfecta (OI) +13C4 relative to WT + 13C4. Treatment with 1D11 corrects RUNX2 expression to WT levels at three‐times weekly dosing for 12 weeks and is dose‐frequency related. Dosing regimen: 3 × wk = three weekly doses; 1 × 2 wk = one dose every other week; 1 × 4 wk = one dose every 4 weeks; all doses 5 mg/kg ip. (*B*) ALP expression, both in trabecular and cortical bone is decreased in OI +13C4 relative to WT +13C4 and is partially restored with 1D11 treatment, short‐term study three‐time weekly dosing, four doses, all doses 5 mg/kg ip. n = 3, 3, and 6 for groups 1–3, respectively.

RUNX2 expression in trabecular bone was significantly increased in OI mice relative to WT mice (Fig. 8*A*). Treatment with 1D11 antibody three times per week decreased the expression to WT levels. As the frequency of dosing was decreased to once every 4 weeks, the expression level of RUNX2 increased back to the levels observed in the 13C4‐treated OI mice.

ALP expression in trabecular and cortical bone was evaluated by immunofluorescence. Although robust expression of ALP was observed in both trabecular and cortical bone of WT mice, expression was very low in OI mice (Fig. 8*B*). Treatment of OI mice with 1D11 antibody three times per week partially restored the expression of ALP. In addition, the morphology of the osteoblasts lining the periosteal surface in OI mice changed to more mature, larger osteoblasts with 1D11 treatment.

**Fig. 9:**
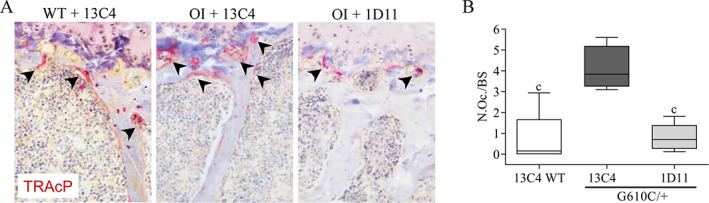
Anti‐TGF‐β antibody 1D11 inhibits hyperactivation of osteoclasts in osteogenesis imperfecta (OI) femoral trabecular bone at three‐times weekly dosing for 12 weeks, 5 mg/kg ip. μCT femur n = 8, 5, and 5 for groups 1–3, respectively. (*A*) OI +13C4 exhibits increased TRACP staining relative to WT +13C4, and this increase is corrected to WT levels with 1D11 treatment. (*B*) Histomorphometry in OI + 13C4 bone exhibits a fivefold increase in N.Oc./BS relative to WT + 13C4, and this increase is corrected with 1D11 treatment to WT levels. Mean ± SD and asterisk(s) denote statistically significant difference compared with OI 13C4. ^c^ *p* < 0.001. N.Oc./BS = the ratio of the number of osteoclasts relative to bone surface area; TRACP = tartrate‐resistant acid phosphatase .

Staining osteoclasts with TRACP marker showed a significant increase in the number of osteoclasts in OI mice when compared with WT littermates (Fig. 9*A*). Treatment with 1D11 antibody three times per week led to a normalization of the number of osteoclasts back to WT mice levels. Supporting the above observations of TRACP staining, OI mice exhibited a profound increase in the number of osteoclasts to bone surface area (N.Oc/BS) compared with WT littermates (Fig. 9*B*). Treatment with 1D11 three times per week led to normalization of N.Oc/BS to WT levels. Cathepsin K expression was also evaluated and was increased in control‐treated OI mice and decreased with 1D11 treatment three times per week (data not shown).

### Biomarkers

**Fig. 10:**
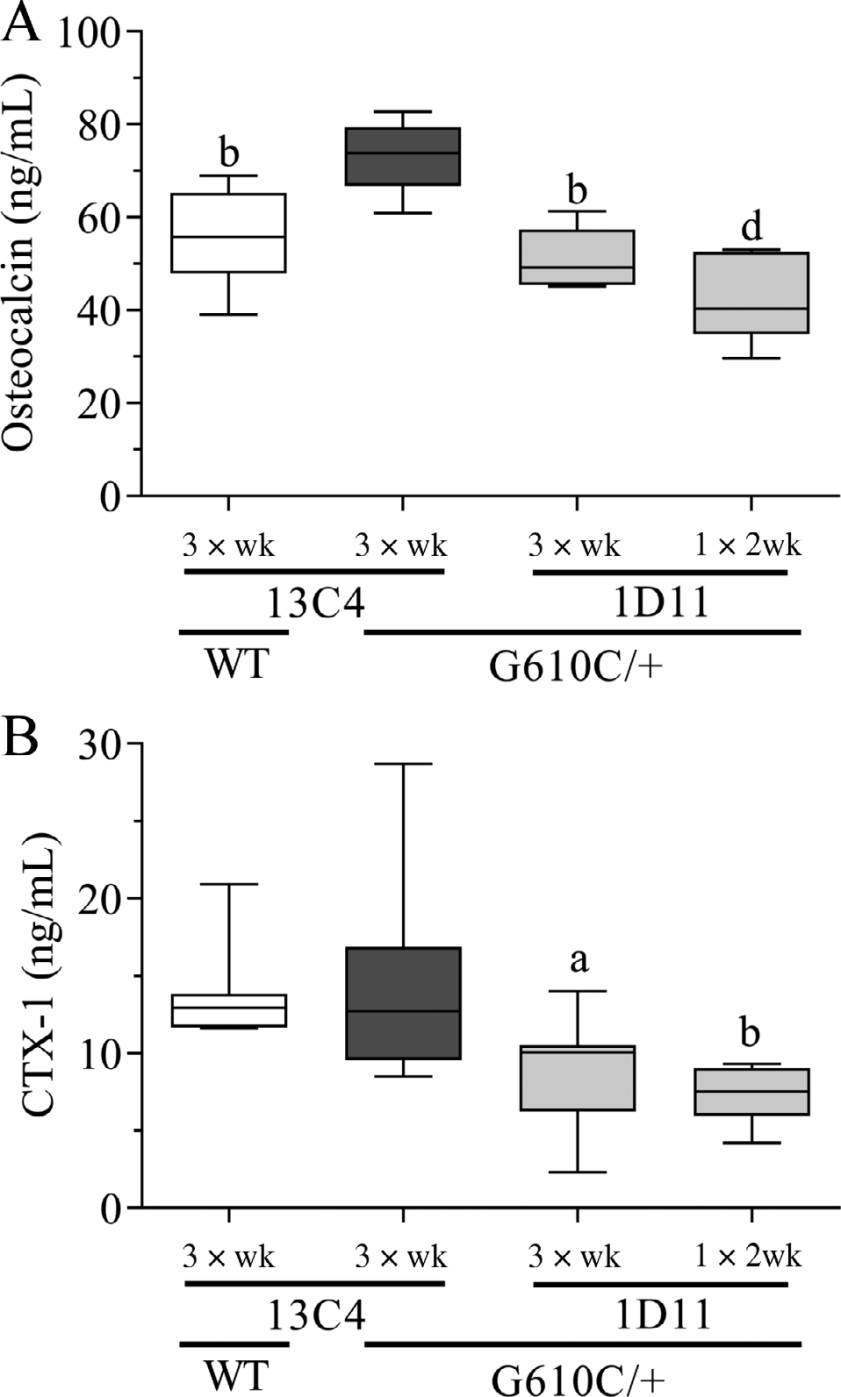
Anti‐TGF‐β antibody 1D11 suppresses serum osteocalcin and C‐terminal telopeptide (CTx‐1) in mice with osteogenesis imperfecta (OI) relative to 13C4 control at dose frequencies including 3 × wk and 1 × 2 wk in OI mice. Osteocalcin is higher in OI compared with WT mice, both treated with 13C4 control, n = 8, 5, 5, and 8 for groups 1–4, respectively. CTx‐1 is not higher in OI compared with WT, n = 10, 10, 10, and 9 for groups 1–4, respectively. Mean ± SD and asterisk(s) denote statistically significant difference compared with OI 13C4, three‐times weekly dosing. ^a^0.01 < *p* < 0.05. ^b^0.001 < *p* < 0.01, ^c^0.0001 < *p* < 0.001, ^d^ *p* < 0.0001. Dosing regimen: 3 × wk = three weekly doses; 1 × 2 wk = one dose every other week; all doses 5 mg/kg ip.

In the dose‐frequency study, OI 13C4‐treated mice exhibited an increase in serum OCN, relative to WT 13C4 mice (Fig. 10*A*). Except for the least‐frequent dosing regimen (once every 4 weeks), all other 1D11 regimens in OI mice significantly suppressed both OCN and CTx‐1 relative to 13C4‐treated OI mice. Additionally, in the same study, OCN levels in OI 13C4‐ and 1D11‐treated mice, significantly (*p* value = 0.0001 and 0.0007, respectively) correlated with both BV/TV and peak force (Data not shown).

## Discussion

The TGF‐β family of growth factors has its major site of residence in bone.^(^ ^5^ ^)^ TGF‐β is produced and secreted in an inactive form, then deposited into the bone matrix.^(^ ^6^, ^7^, ^8^ ^)^ During resorption, osteoclasts release and activate TGF‐β from the bone matrix, freeing this bioactive molecule to influence the bone microenvironment.^(^ ^9^ ^)^ TGF‐β also couples bone resorption and formation and recruits osteoprogenitors to the site of bone resorption.^(^ ^10^ ^)^

Direct, disease‐specific evidence for increased TGF‐β signaling in OI has been generated. A study in type III patients with OI showed upregulation of TGF‐β signaling, highlighting this protein's central role in the pathogenesis of OI.^(^ ^11^ ^)^ Types III and IV patients exhibited increased phospho‐SMAD2 staining in bone sections, supporting the preclinical finding of increased TGF‐β activity in OI bone.^(^ ^12^ ^)^ In fact, regulation of SMAD phosphorylation was the most significantly upregulated molecular event, whereas TGF‐β activation was identified as the most significant upstream regulator.

TGF‐β inhibition, using the mouse anti‐TGF‐β monoclonal antibody 1D11, has been shown to increase bone volume in vivo in WT and several mouse models of disease. These have included both a model of chronic kidney disease^(^ ^13^ ^)^ and a model of basal‐like breast cancer metastasis.^(^ ^24^ ^)^ Nyman et al. showed that TGF‐β inhibition in mouse models of myeloma, exhibiting increased TGF‐β signaling, resulted in increased trabecular bone volume, improved trabecular architecture, and increased vertebral body strength in biomechanical compression tests.^(^ ^25^ ^)^ Grafe et al. administered 1D11 for 8 weeks, showed increased BV/TV to levels close or equivalent to WT levels in both the CRTAP and G610C OI mouse.^(^ ^12^ ^)^ In addition, femoral strength was increased in the CRTAP mice, even though no changes were observed in diaphyseal cortical bone. No biomechanical results were reported for vertebra in either model or femur in the G610C mice. These authors also showed upregulation of TGF‐β–related genes, *Cdkn1a* and *Serpine*, and increased phosphorylation of SMAD2, in both the CRTAP and G610C OI mouse. Additional support for a potential disease‐specific mechanism was observed by Yang et al. when using 1D11 to treat an E‐selectin ligand‐1 KO (*Esl1*−/−) mouse that had increased TGF‐β activity and severe osteopenia.^(^ ^6^ ^)^ In this case, after 1D11 treatment, *Esl1*−/− mice exhibited markedly higher bone volume. These last two examples reinforce that increased TGF‐β signaling in OI is a potential target for disease modification.

In contrast, Tauer et al. reported that 1D11 was mostly ineffective in the *col1A* ^*Jrt+*^ mouse model.^(^ ^20^ ^)^ This model suffers from spontaneous fractures, a trait that it shares with severe patients with OI in the clinic, making it attractive for translational research. However, there are some characteristics of the *col1A* ^*Jrt+*^ mouse that may point to a genotype‐specific effect when inhibiting the TGF‐β pathway. The *col1A* ^*Jrt+*^ mouse has no corresponding human OI mutation and includes facets of a different disease (Ehlers‐Danlos syndrome) that affects connective tissue, primarily the skin, joints, and blood vessel walls.^(^ ^25^ ^)^ The *col1A* ^*Jrt+*^ mouse model also exhibits a dentin defect, which may affect feeding, thus having the real potential to cause nutritional deficiencies that could affect bone health.^(^ ^20^ ^)^ This contrasts with the two mouse models described in this article, the G610C, which was produced by inserting a mutation from an already known population of Amish patients with OI, and the CRTAP KO, which genetically represents OI type VII.^(^ ^26^ ^)^ In spine and femoral neck, children with OI (types I, III, and IV) had BMD values that were 62.6% and 63.0% of healthy patients, respectively.^(^ ^27^ ^)^ The G610C model with BV/TV relative to WT ranging from 44% to 65% and 63% to 73% for lumbar and femur, respectively, translates to what is observed in patients with OI. And last, pharmacologically speaking, similar to the results of anti‐TGF‐β treatment in the *col1A* ^*Jrt+*^ mouse model, antisclerostin‐antibody treatment had very little effect. This therapy has exhibited efficacy in at least two OI mouse models and is currently in clinical trials for OI.^(^ ^28^ ^)^ Although the above arguments do not address the fact that the G610C mouse rarely suffers from spontaneous fractures like the *col1A* ^*Jrt+*^, we believe that the G610C model is appropriate for investigating new therapeutics for OI and the differences in efficacy with the TGF‐β antibody observed in the different mouse models might point to different effects of the antibody in different OI types.

Our data have profoundly increased our understanding of OI and the response to TGF‐β inhibition using 1D11. Serum bone biomarkers were suppressed by 1D11 administration, building on observations made by Tang et al., who suggested that inhibition of the TGF‐β pathway with a TGF‐β type I receptor inhibitor restored the coupling between osteoblasts and osteoclasts.^(^ ^10^ ^)^ BFR/BS and MAR were decreased with 1D11 treatment (Fig. 7), an observation shared by Liu et al., where 1D11 decreased both in a dose‐responsive fashion in a high‐turnover renal osteodystrophy model.^(^ ^13^ ^)^ Suppression of RUNX2, CTx‐1, and OCN by 1D11 with a corresponding increase in ALP suggests an effect of the antibody on bone turnover. Moreover, evaluation of the l6 vertebrae, with enhancement of BV/TV in the dominant OI mouse, and other trabecular parameters after 1D11 treatment confirms the previous observations by Grafe et al.^(^ ^12^ ^)^ However, critically we show that these improvements lead to a profound increase in the strength of the lumbar bone. Finally, an evaluation of the biomechanical attributes of the femur in the more common dominantly inherited OI mouse showed enhancement of peak force, bending strength, yield force, and yield strength. Ct.Ar increased at the three highest frequencies tested and trended upward in the dose‐response study. Trends for increases in Ct.Th were also noted in both the dose‐response and frequency studies. We have previously shown in the *jck* mouse, a high bone‐turnover model, increases in Ct.Th with 1D11 treatment.^(^ ^13^ ^)^ It is possible that these small changes in cortical parameters after treatment with 1D11, led to the biomechanical enhancement observed. Future work will be needed to further determine the mechanism for this effect.

### Inhibition of TGF‐β modulates expression of bone markers

In OI mice we have observed increased levels of RUNX2 and TRACP staining in the trabecular bone, along with increased OCN in the serum, whereas ALP levels were decreased in trabecular and cortical bone, relative to WT mice. 1D11 led to dose‐ and frequency‐dependent decreases in RUNX2 expression, serum OCN, and CTx‐1. RUNX2 is known as the predominant regulator of osteoblast differentiation, gene expression, and function. Although RUNX2 acts in a positive manner at early stages of osteoblast differentiation later it is inhibitory.^(^ ^29^ ^)^ SMADs also have been shown to interact with RUNX2 by increasing its transcriptional activity.^(^ ^30^ ^)^ OI mice have increased SMAD2 activation,^(20^ ^)^ and this may be acting to maintain high levels of RUNX2 in the trabecular bone, thus impairing osteogenesis.

An analysis of bone sections of patients with OI found reduced level and/or altered activity of ALP—suggesting dysfunctional osteoblasts.^(^ ^31^, ^32^ ^)^ Consistent with these localization studies in patients with OI, we have found lower ALP in lumbar trabecular bone in OI mice. In addition, an increase in expression was observed upon 1D11 treatment. Further studies are required to better understand the specific effects of TGF‐β inhibition on osteoblast function in the setting of OI.

Evidence of increased bone turnover has been observed in patients with OI.^(^ ^33^, ^34^ ^)^ Histomorphometrically, young patients with OI exhibit high bone turnover, increased osteoblast and osteoclast surfaces, increased bone formation rate, and decreased MAR.^(^ ^33^ ^)^ Li et al. showed that impaired differentiation of osteoblasts contributed to the pathophysiology of OI, by secreting factors known to influence increase osteoclast number and function.^(^ ^35^ ^)^ Tang et al. observed in in vitro and in vivo models that active TGF‐β1 released during bone resorption coordinates bone formation by inducing migration of bone mesenchymal stem cells, to the bone resorptive sites and that this process is mediated through a SMAD signaling pathway.^(^ ^10^ ^)^ In our mouse model, we saw evidence of increased osteoclast number via increased TRACP staining and number of osteoclasts per bone surface in trabecular bone. 1D11 reduced TRACP expression and osteoclast numbers to WT levels. The observation of reduced bone formation (BFR/BS and MAR)—with 1D11 treatment in our mouse model with robust increases in vertebral volume and strength—adds additional evidence of the effect of the TGF‐β antibody on bone turnover.

### TGF‐β inhibition increases bone strength in an OI mouse model

Little et al. administered zoledronic acid (once weekly at 0.025 mg/kg), a commonly prescribed bisphosphonate in OI, from 5 to 9 weeks of age in the G610C mouse model.^(^ ^36^ ^)^ The authors collected the third lumbar bone and tibia for biomechanical testing. With the caveat of different age, length of treatment of 4 weeks compared with our studies of 8 to 12 weeks of treatment and the use of the tibia versus femur, a comparison can be made. For the lumbar bone, zoledronic acid led to a 44% increase in biomechanical strength, whereas 1D11 led to 101% to 129% increase in biomechanical strength.^(^ ^36^ ^)^ For the tibia, the authors observed a biomechanical strength increase of 22%, whereas 1D11 led to an increase between 14% and 31% in the femur.^(^ ^36^ ^)^ Bisphosphonates have proven their effectiveness in both osteoporosis and OI, at least when used short term, a benefit to patients with OI including decreased fracture risk and increases to quality of life has been shown, mostly in pediatric patients.^(^ ^37^ ^)^ A retrospective study reviewed fracture imaging of a bisphosphonate‐treated compared with a historical group of patients with OI before bisphosphonate use in an attempt to identify the locations of femoral fractures.^(^ ^38^ ^)^ In each cohort, the majority^(^ ^6^ ^)^ of fractures in the bisphosphonate group were in the subtrochanteric region, whereas the control group exhibited a more widespread fracture pattern, with the majority in the diaphyseal region. Again, the authors pointed out that bisphosphonates are efficacious but possibly high bending moments in the proximal femur and the aforementioned suppression of the homeostatic effect of bone remodeling, atypical fractures occur at a higher rate with long‐term bisphosphonate treatment necessitating modification of surgical protocols of such patients. A recent study suggests that atypical diaphyseal fractures are more likely associated with bone deformation in patients with OI rather than treatment with bisphosphonates.^(^ ^39^ ^)^

Our studies have found that exogenous administration of 1D11, an anti‐TGF‐β antibody, enhances biomechanical attributes of bone in OI mice. This response was dose and frequency dependent. The minimum effective dose was 0.5 mg/kg and the minimum effective dose frequency was 1 × 4 weeks. Robust dose‐frequency–related efficacy was maintained at least out to 1 × 2 weeks, which coincides closely with mouse bone remodeling time of approximately 2 weeks.^(^ ^40^ ^)^ Encouragingly, human bone remodeling time ranges from 6 to 9 months,^(^ ^41^ ^)^ suggesting that infrequent dosing may be possible in patients with OI with this therapy.

Importantly, the effect size observed in this study was large, especially in trabecular bone. Maximum percentage change in peak force (biomechanical strength) observed following 1D11 administration in the dose‐response and dose‐frequency study for the vertebrae was 129% and 101%, respectively, above control‐treated OI mice; this translated to 26% and 19% above WT values. This profound increase in vertebral bone strength (and volume) has great potential for patients with OI, who all too often suffer from painful and debilitating compression fractures of the spine. Additionally, in our study, we observed increases following 1D11 administration in peak force, bending strength, yield force, and yield strength to WT levels, which is consistent with biomechanical data reported in Grafe et al.^(^ ^12^ ^)^ For peak force (strength), the percentage increase in the femur following 1D11 administration in the dose response and dose frequency study was 14% and 31%.

Our studies show that blocking TGF‐β activity with an anti‐TGF‐β antibody in the dominant OI mouse model leads to profound efficacy in the lumbar and femoral bones measured via imaging and biomechanical testing. We report several important mechanisms underlying these promising findings, including suppression of osteoclast function and decreased bone turnover as assessed by bone histomorphometry. Our results and others suggest that anti‐TGF‐β therapy leads to the restoration of normal bone turnover. Taken together, these data suggest a potential for a standard of care therapy for a disease without an approved treatment that has the potential to enhance the lives of patients with OI around the world.

## Conflict of Interest

The authors all declare there is no conflict of interest regarding the publication of this article. All authors, unless stated otherwise, are employees of Sanofi. Oxana Beskrovnaya and Ryan Russo are currently employed with Dyne Therapeutics, Shannon Dwyer with Artax Biopharma, Nikolai Bukanov with Janssen Pharmaceuticals, Jeffry Nyman and Sasidhar Uppuganti with Vanderbilt University, and Yves Sabbagh with Inozyme Pharma. Yves Sabbagh is also a shareholder of Sanofi.

### Peer Review

The peer review history for this article is available at https://publons.com/publon/10.1002/jbm4.10530.

## Supporting information

**Supplementary Figure S1** Anti‐TGF‐β antibody 1D11 induces robust and durable dose frequency related increases in lumbar bone (l4) trabecular parameters in OI mice, measured by histomorphometry. (*A*) Bone volume, (*B*) trabecular separation, (*C*) trabecular number, and (*D*) trabecular thickness of OI and WT mice. Mean ± SD, asterisk(s) denote statistically significant difference compared with OI 13C4, 3 × wk (^a^0.01 < *p* < 0.05, ^b^0.001 < *p* < 0.01, ^c^0.0001 < *p* < 0.001, ^d^ *p* < 0.0001). Dosing regimen abbreviations: 3 × wk (3 weekly doses), 1 × wk (one weekly dose), 1 × 2wk (one dose every other week), 1 × 4wk (one dose every 4 weeks), all doses 5 mg/kg, ip n = 8, 5, 5, 8, 8, and 8 Groups 1–6, respectively.
