# Supplementary figures and images for: Inhibition of TGF‐β Increases Bone Volume and Strength in a Mouse Model of Osteogenesis Imperfecta
Source: JBMR Plus. 2021 Aug 3;5(9):e10530. doi: 10.1002/jbm4.10530 (PMC8441395; doi:10.1002/jbm4.10530)

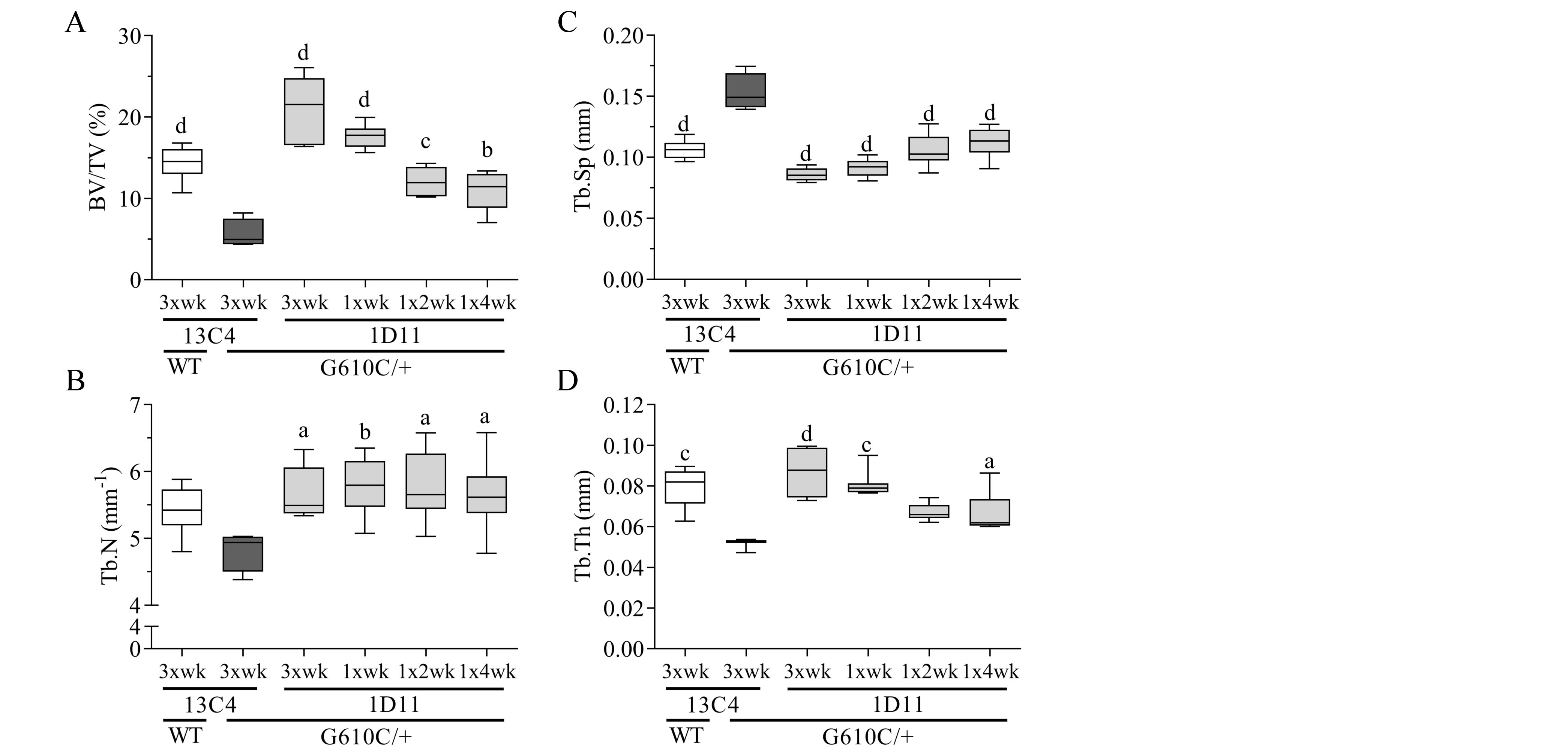

Supplement: Supplementary file 1 — Supplementary Figure S1 Anti‐TGF‐β antibody 1D11 induces robust and durable dose frequency related increases in lumbar bone (l4) trabecular parameters in OI mice, measured by histomorphometry. (A) Bone volume, (B) trabecular separation, (C) trabecular number, and (D) trabecular thickness of OI and WT mice. Mean ± SD, asterisk(s) denote statistically significant difference compared with OI 13C4, 3 × wk (a0.01 < p < 0.05, b0.001 < p < 0.01, c0.0001 < p < 0.001, d p < 0.0001). Dosing regimen abbreviations: 3 × wk (3 weekly doses), 1 × wk (one weekly dose), 1 × 2wk (one dose every other week), 1 × 4wk (one dose every 4 weeks), all doses 5 mg/kg, ip n = 8, 5, 5, 8, 8, and 8 Groups 1–6, respectively. [file JBM4-5-e10530-s001.tif]
